# Evaluating the effect of grape syrup on iron deficiency anemia in women: A randomized clinical trial

**DOI:** 10.1002/fsn3.4185

**Published:** 2024-05-06

**Authors:** Rasul Pourhakim, Fatemeh Emadi, Saeed Razavi Dizaji, Daryush Talei, Sayed Saeed Esmaeili Saber, Maryam Iranzadasl

**Affiliations:** ^1^ Department of Traditional Medicine, School of Persian Medicine Shahed University Tehran Iran; ^2^ Traditional Medicine Clinical Trial Research Center Shahed University Tehran Iran; ^3^ Department of Internal Medicine, Faculty of Medicine Urmia University of Medical Sciences Urmia Iran; ^4^ Medicinal Plants Research Center Shahed University Tehran Iran

**Keywords:** anemia, grape syrup, iron deficiency anemia

## Abstract

Globally, iron deficiency reigns as the most prevalent nutritional disorder, with anemia disproportionately impacting women of childbearing age. Despite the effectiveness of existing treatments, like iron supplements, their side effects remain a concern. This study explores the potential of grape syrup (GS), a functional iron‐rich food, to modulate markers of iron‐deficient anemia in women. A randomized, double‐blind study explored the impact of GS on iron deficiency anemia markers in 130 women. Participants were allocated to intervention or placebo groups. For 4 weeks, the intervention group received a daily 50 mg ferrous sulfate tablet alongside 10 cc of GS thrice daily. The placebo group received a 50 mg ferrous sulfate tablet with a 10 cc placebo syrup thrice daily. Before and after the intervention, key markers like red blood cell count (RBC), mean corpuscular volume (MCV), hemoglobin (Hb), hematocrit (Hct), ferritin, total iron binding capacity (TIBC), and serum iron were assessed. Notably, both Hb and Hct levels were significantly higher in the GS group (*p* < .05). Additionally, RBC and MCV values showed significant improvement compared to the placebo group (*p* < .05). However, no significant difference was observed for other iron deficiency markers like serum iron, ferritin, and TIBC (*p* > .05). This study's findings suggest that combining grape syrup with iron tablets might offer potential advantages over iron tablets alone in managing iron deficiency anemia.

## Graphical abstract

This randomized, double‐blind clinical trial was achieved for the assessment of the grape molasses (GM) effect on iron deficiency anemia in women aged 15–49 years. In a 4‐week intervention, GM with ferrous sulfate tablets increased hemoglobin, hematocrit, red blood cell count, and mean corpuscular volume significantly more than placebo syrup with ferrous sulfate tablets.

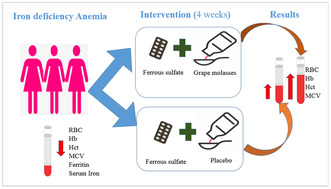

## 1 INTRODUCTION

Globally, anemia affects a staggering one‐third of the population, with prevalence rising with age and disproportionately impacting women of childbearing age, pregnant women, and older adults (Cappellini et al., 2020). In particular, heavy menstrual bleeding affects roughly 30% of women of childbearing age, increasing their risk of iron deficiency anemia (IDA) (Mansour et al., 2021). The World Health Organization categorizes IDA as a major public health burden with significant economic costs (Newhall et al., 2020). Beyond its economic impact, IDA can lead to various health complications, including recurrent infections, digestive issues, obesity, and gum disease (Cappellini et al., 2020). However, it is associated with gastrointestinal side effects such as constipation, bloating (Bloor et al., 2021), and an increase in the number of pathological bacteria in the colon that can lead to inflammatory diseases and colon cancer (Tolkien et al., 2015). This underscores the urgent need for safe and effective alternative treatments to address this widespread health concern.

The role of nutrition in managing IDA has gained significant attention in recent years (Newhall et al., 2020; Zulfiqar et al., 2021). Traditional Persian Medicine (TPM) emphasizes the importance of dietary interventions for both the prevention and treatment of various ailments (Avicenna, 2005; Bahaeddin et al., 2023; Nimrouzi & Zare, 2014). Notably, TPM recommends various foods like meat, eggs, figs, grapes, and grape syrup for addressing anemia (Avicenna, 2005; Fatali et al., 2020). Grape syrup, a delicious and nutritious food typically enjoyed at breakfast with milk or sesame, is a popular processed product derived from grapes. It features a dark color, a sweet taste, and a dense consistency. Traditionally produced for centuries by boiling and condensing grape juice (Helvacioglu et al., 2018), it has recently gained traction in the food industry as a natural sweetener alternative to refined sugar (Levent et al., 2022; Mousavi Kalajahi, 2021).

Grape syrup boasts a rich nutritional profile, comprising high levels of glucose, fructose, organic acids, and essential minerals like iron, copper, zinc, calcium, and magnesium (Helvacioglu et al., 2018; Tavakolipour et al., 2020). This mineral content, coupled with its easy digestibility, allows grape syrup to meet up to 37% of daily iron needs (Aşcı & Baydar, 2021). Additionally, it possesses antioxidant, anti‐mutagenic, anticancer, and anti‐inflammatory properties (Pérez‐Ortiz et al., 2019; Wang et al., 2011). Given its potential, this study investigated the effect of grape syrup on hematological markers of IDA (hemoglobin, hematocrit, red blood cell count, mean corpuscular volume, ferritin, and serum iron) in women with the condition. We hypothesized that grape syrup supplementation in women with IDA would significantly improve key hematological markers of the condition, including hemoglobin, hematocrit, red blood cell count (RBC), mean corpuscular volume (MCV), ferritin, and serum iron.

## 2 MATERIALS AND METHODS

### 2.1 Participants and study setting

This randomized clinical trial, conducted at Imam Khomeini Hospital (Urumia, Iran) between February 2020 and October 2022, included women aged 15–49 with diagnosed anemia (Hgb 10–12 confirmed by a hematologist). Exclusion criteria comprised systemic/chronic diseases, abnormal bleeding (GI, menorrhagia, hematuria), recent delivery (<2 months), recent surgery (2–3 months), thalassemia, tuberculosis, sickle cell disease, pregnancy, diabetes, grape/product allergies, and iron pill allergies.

Additional exclusion criteria included pregnancy during the study, severe complications and allergic reactions (e.g., hematuria, urticaria), participant withdrawal, consumption of other drugs, and medication changes. The study was approved by the Shahed University IRB (code: IR.SHAHED.REC.1398.108) and registered at the Iranian Registry of Clinical Trials (https://irct.ir/code:IRCT20200120046205N1).

### 2.2 Study design

#### 2.2.1 Preparation of grape syrup and placebo

Locally sourced grape syrup (GS), prepared with white grapes, was obtained in 250‐mL dark glass bottles. Patients received a 4‐week supply (four bottles) at the prescribed dosage.

A placebo, matching the consistency and color of GS, was prepared using sodium saccharin (Merck, Germany), carboxymethyl cellulose (Henzak Chemi Co., Iran), and caramel (Shirinkar Co., Iran). Both GS and placebo were stored in identical 250‐mL dark glass bottles. Iron tablets containing 50 mg of ferrous sulfate were sourced from Darupakhsh Chemical Co., Iran.

#### 2.2.2 Randomization, blinding, and intervention

Eligible women were randomly assigned (1:1) to intervention or placebo groups using a computer‐generated table. Allocation was concealed via serially numbered opaque envelopes. A separate computer program assigned unique codes to each drug/placebo, included on labels with consumption instructions. Both researchers and participants remained blinded to the codes.

For 4 weeks, the intervention group received 50 mg of ferrous sulfate tablets orally and 10 cc of grape syrup in lukewarm water thrice daily (1 h before meals). The control group received the same dose of ferrous sulfate but with 10 cc placebo syrup instead of grape syrup, also administered thrice daily, 1 h before meals.

### 2.3 Outcomes

Both groups underwent pre‐ and post‐intervention assessments of vital signs (pulse, respiration, blood pressure). Both groups underwent pre‐ and post‐intervention assessments of key hematological markers for anemia (Hb, Hct, MCV, RBC, serum iron, ferritin, and TIBC). The primary outcome was the change in Hb concentration over the 4‐week intervention.

Secondary outcomes included changes in other laboratory markers (HCT, MCV, RBC, serum iron, ferritin, TIBC, AST, and ALT), vital signs, adverse effects, participant dropout rates, and quality of life.

Both groups participated in quality‐of‐life assessments using the validated Persian translation of the General Health Questionnaire‐28 (GHQ‐28) at baseline and after 4 weeks. Researchers have previously established the validity and reliability of this questionnaire in the Iranian context (Nazifi et al., 2014). A dedicated questionnaire specifically designed to record potential side effects was used to monitor for any adverse reactions throughout the study.

GS was analyzed for heavy metals, including iron (Fe), copper (Cu), zinc (Zn), lead (Pb), arsenic (As), and tin (Sn), using flame atomic absorption spectrometry (AA‐670 Shimadzu, Japan). Permissible limits for these metals were based on the Iranian National Standards Organization (INSO) index (Heshmati et al., 2020; INSO, 2012) (Table 4).

### 2.4 Sample size

The sample size was determined using a superiority analysis assuming normally distributed data, a 5% type I error, a two‐sided test, and 80% power. We considered a minimum of 40 participants per group based on previous studies (Powers et al., 2017; Shokri & Ali, 2022).

### 2.5 Data analysis

Data analysis was conducted using SPSS (version 26). Quantitative data were presented as mean ± standard deviation (SD), while qualitative data were reported as frequencies and percentages. Between‐group comparisons were performed using independent t‐tests for normally distributed data and Mann–Whitney *U*‐tests for non‐normally distributed data. All reported *p*‐values were interpreted at a significance level of *p* < .05.

## 3 RESULTS

**FIGURE 1:**
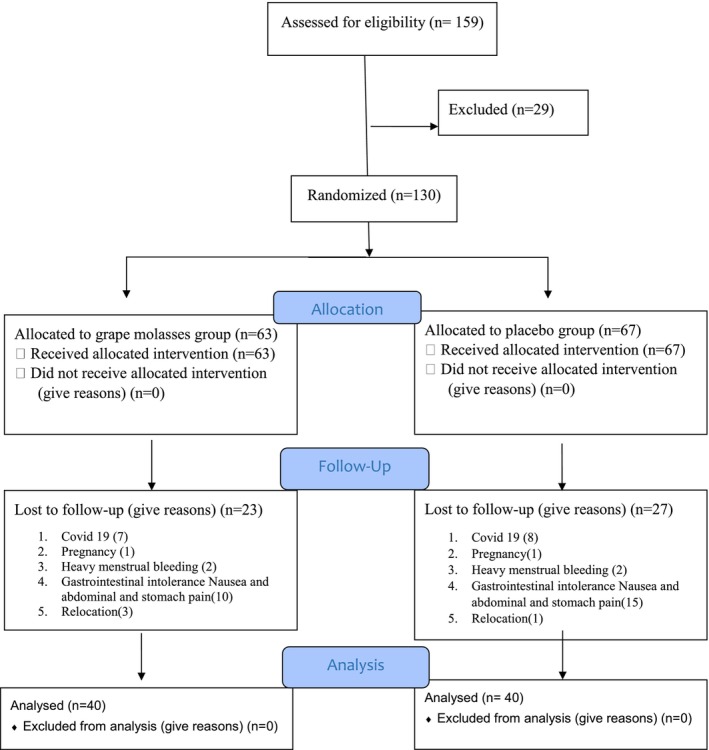
Participant flowchart.

Initially, 159 women with IDA expressed interest in the study. After screening for eligibility and availability, 130 were enrolled and randomly assigned to either the grape syrup (*n* = 63) or placebo (*n* = 67) group. However, 50 participants dropped out throughout the study (23 in the GS group and 27 in the placebo group), as shown in Figure 1. Ultimately, 80 participants completed the study and were analyzed (40 in each group) with similar baseline characteristics across both groups (Table 1).

**TABLE 1:** Baseline characteristics of women with iron deficiency anemia.

Characteristics	Ferrous sulfate (50 mg orally) + grape syrup group (*n* = 40)	Ferrous sulfate (50 mg orally) + placebo group (*n* = 40)	*p‐*Value
Age (years)	36.15 ± 8.17	34.67 ± 8.27	.80
Education no (%)			
None	6 (15%)	9 (22.5%)	.38
<High school	18 (45%)	17 (42.5%)	
High school	10 (25%)	11 (27.5%)	
University	6 (15%)	3 (7.5%)	
Occupation, no (%)			
Housekeeper	33 (82.5%)	35 (87.5%)	.53
Employee	7 (7.5%)	5 (12.5%)	
Income			
Low	24 (60%)	21 (52.5%)	.50
Medium	16 (40%)	19 (47.6%)	
PR (beat per minute)	81.30 ± 7.06	81.40 ± 8.31	.95
RR (breath per minute)	19.75 ± 4.58	19.35 ± 3.19	.65
BP (mmHg)	113.24 ± 9.69	115.23 ± 12.37	.43
AST (U/L)	22.52 ± 6.52	23.62 ± 10.49	.57
ALT (U/L)	17.38 ± 5.93	20.13 ± 13.02	.22
ALP (U/L)	197.21 ± 74.97	204.60 ± 78.04	.66
RBC (million/mm^3^)	4.49 ± 0.06	4.49 ± 0.06	.09
Hb (g/dL)	10.70 ± 0.09	10.91 ± 0.08	.08
Hct (%)	35.77 ± 2.42	36.50 ± 1.73	.12
MCV (fL)	79.07 ± 5.54	80.85 ± 6.06	.17
Fe (ng/mL)	22.61 ± 18.45	26.96 ± 17.78	.28
TIBC (mcg/dL)	447.82 ± 74.93	430.28 ± 96.98	.36
Ferritin (ng/mL)	8.66 ± 6.40	11.35 ± 7.03	.10
GHQ 28	62.85 ± 16.61	61.07 ± 11.58	.58

*Note*: Data are expressed as mean ± standard deviation. Statistical significant difference (*p* < .05).

Abbreviations: ALP, alkaline phosphatase; ALT, alanine aminotransferase; AST, aspartate aminotransferase; BP, blood pressure; fL, femtoliters; g/dL, grams per deciliter; GHQ 28, General Health Questionnaire 28; Hb, hemoglobin; Hct, hematocrit; mcg/dL, micrograms per deciliter; MCV, mean corpuscular volume; million/mm^3^, millions per cubic millimeter; mmHg, millimeters of mercury; ng/mL, nanograms per milliliter; PR, pulse rate; RBC, red blood cell; RR, respiratory rate; TIBC, total iron binding capacity; U/L, units per liter.

Both groups showed significant improvements in key hematological markers after 4 weeks of intervention (Table 2). Notably, the red blood cell count (RBC) increase was significantly greater in the GS group (0.29 ± 0.30) compared to the placebo group (0.11 ± 0.03) (*p* = .003). Similarly, the MCV increase was significantly higher in the GS group (4.13 ± 0.67) compared to the placebo group (1.47 ± 0.48) (*p* = .002). These findings suggest that the GS intervention led to greater improvements in RBC and MCV compared to placebo.

**TABLE 2:** Comparison of hematological indices in women with iron deficiency anemia at baseline visit and at 4 weeks after treatment within and between groups.

Variable	Ferrous sulfate + grape syrup group (*n* = 40)		*p*‐Value (effect size)	Ferrous sulfate + placebo group (*n* = 40)		*p*‐Value (effect size)	Between 2 groups after treatment *p*‐value (effect size)	Mean deference between 2 groups *p*‐value
	Before	After		Before	After			
RBC (million/mm^3^)	4.49 ± 0.06	4.78 ± 0.37	.001* (.753)	4.49 ± 0.06	4.61 ± 0.43	.001* (.310)	.059 (.395)	.003*
Hb (g/dL)	10.70 ± 0.09	13.30 ± 0.76	.018* (3.454)	10.91 ± 0.08	12.16 ± 0.51	.013* (2.475)	.001* (1.795)	.049*
Hct (%)	35.77 ± 2.42	40.40 ± 3.21	.027* (1.368)	36.50 ± 1.73	37.82 ± 1.75	.001* (.642)	.0001* (.769)	.0001*
MCV (fL)	79.07 ± 5.54	83.20 ± 4.88	.000* (1.105)	80.85 ± 6.06	82.32 ± 5.48	.001* (.357)	.45 (.111)	.002*
Fe (ng/mL)	22.61 ± 18.45	44.85 ± 28.97	.005* (.910)	26.96 ± 17.78	41.95 ± 23.21	.001* (.744)	.622 (.071)	.146
TIBC(mcg/dL)	447.82 ± 74.93	416.44 ± 73.15	.000* (.445)	430.28 ± 96.98	411.07 ± 78.35	.046* (.225)	.752 (.070)	.487
Ferritin (ng/mL)	8.66 ± 6.40	18.32 ± 8.75	.001* (1.172)	11.35 ± 7.03	22.39 ± 11.22	.001* (1.129)	.075 (.283)	.58

*Note*: Data are expressed as mean ± standard deviation.

Abbreviations: fL, femtoliters; g/dL, grams per deciliter; Hb, hemoglobin; Hct, hematocrit; mcg/dL, micrograms per deciliter; MCV, mean corpuscular volume; ng/mL, nanograms per milliliter; RBC, Red blood cell; TIBC, total iron binding capacity.

* Statistical significant difference (*p* < .05).

Hemoglobin and hematocrit levels showed significantly higher increases in the GS group compared to the placebo (*p* = .0001), suggesting a more substantial improvement in these key anemia markers (Table 2). However, no significant differences were observed for iron, ferritin, or TIBC between the groups after the intervention.

Quality of life, as measured by the GHQ‐28, did not differ significantly between the GS and placebo groups after the intervention. However, the GS group showed a statistically significant improvement in GHQ‐28 scores compared to the baseline (*p* = .001), suggesting an overall positive impact on quality of life. No significant changes were observed in vital signs or liver function tests in the intervention group compared to the placebo (*p* > .1) (Table 3). The analysis of heavy metals in the grape syrup confirmed levels within the acceptable ranges set by the Iranian Standard Organization (Table 4).

**TABLE 3:** Comparison of vital signs and liver tests between two groups after intervention.

Characteristics	Ferrous sulfate (50 mg orally) + grape syrup group(*n* = 40)	Ferrous sulfate (50 mg orally) + placebo group (*n* = 40)	*p*‐Value
PR (beat per minute)	77.67 ± 4.71	76.37 ± 5.30	.251
RR (breath per minute)	18.92 ± 3.61	18.82 ± 2.40	.885
BP (mmHg)	112.73 ± 8.13	112.17 ± 18.12	.859
AST (U/L)	21.37 ± 6.38	23.39 ± 10.26	.294
ALT (U/L)	18.42 ± 7.42	21.05 ± 13.07	.272
ALP (U/L)	190.20 ± 68.81	192.01 ± 79.84	.914

Abbreviations: ALP, alkaline phosphatase; ALT, alanine aminotransferase; AST, aspartate aminotransferase; BP, blood pressure; mmHg, millimeters of mercury; PR, pulse rate; RR, respiratory rate; U/L, units per liter.

**TABLE 4:** Concentration of heavy metals in grape syrup.

Parameter	Symbol	Dose (mg/L)	Maximum permissible levels (milligrams per kilogram)^a^
Lead	Pb	0.05	0.3
Arsenic	As	0.02	0.2
Tin	Sn	3.1	150
Copper	Cu	0.5	5
Zinc	Zn	0.2	5
Iron	Fe	7.1	25

^a^ The permissible limits were determined based on Iranian National Standards Organization (INSO) index.

Both groups reported mild gastrointestinal side effects, including nausea, abdominal pain, and stomach discomfort. However, the incidence was slightly higher in the placebo group (15) compared to the grape syrup group (10). No severe side effects were observed in either group.

## 4 DISCUSSION

The study found significant improvements in key blood parameters for anemia after the intervention in the GS group compared to the placebo (*p* < .003). This included higher increases in hemoglobin, hematocrit, red blood cell count, and MCV in the GS group. Additionally, quality of life, as measured by the GHQ‐28 score, significantly improved in the GS group (*p* = .001). Intriguingly, serum iron, ferritin, and total iron‐binding capacity (TIBC) did not differ significantly between groups, suggesting these markers may require longer observation periods (3–6 months) to reflect changes after oral iron supplementation, as noted in previous studies (Camaschella, 2015; Ning & Zeller, 2019).

Grape syrup, a popular food product globally, is particularly common at breakfast in many Middle Eastern countries, often paired with sesame paste. It also finds application in desserts and cookies as a sugar substitute to enhance taste (Özmen et al., 2023; Rahbari et al., 2023). Its high carbohydrate content makes it a good source of energy, while its mineral compounds, phenolic content, and organic acids contribute to its functional food status (Karaman et al., 2017). Notably, iron (Fe) in grape syrup demonstrates high digestibility and absorption by the digestive system (Aşcı & Baydar, 2021). A study involving 56 children aged 6–36 months compared iron absorption from grape syrup and ferrous sulfate, concluding that grape syrup could be an effective iron source for preventing iron deficiency anemia in children (Aslan et al., 1997).

Grapes are a significant fruit source of valuable nutrients, including antioxidants, antimicrobials, and anticancer compounds (Soleymanfallah et al., 2022). Incorporating various grape products into daily diets undoubtedly plays a crucial role in promoting healthy food habits and overall well‐being (Aşcı & Baydar, 2021). Grape juice, in particular, boasts high levels of glucose, fructose, sucrose, vitamins A, C, B1, B2, minerals, organic acids, and antioxidant agents (Rezaei et al., 2020). This makes it a valuable dietary component for individuals of all ages, especially children, women, and athletes (Hatamikia et al., 2013). This study aimed to determine the macro‐ and microelement content of a specific grape product known as grape shinny (molasses).

This study revealed a high concentration of essential minerals in grape syrup, including iron, zinc, calcium, potassium, and copper. Levels of toxic elements were minimal, confirming their safety for human consumption and potential benefits for child development (Rasulov et al., 2020). However, concerns exist regarding the heavy metal content in food products like grape syrup (Krarapina & Kilicel, 2020). Our analysis found heavy metal concentrations within acceptable limits set by the Iranian Standard Organization, further supporting the safety and healthfulness of grape shinny as a food source.

Heavy metals fall into two categories: essential and non‐essential. Non‐essential and hazardous metals like Hg, Pb, Cr, Cd, and As are well known for inducing human poisoning. Essential heavy metals like Cu, Fe, Mn, Co, and Zn, on the other hand, are crucial for various biochemical and physiological functions (El Ati‐Hellal & Hellal, 2021). Finally, due to high consentrarion of essential mierals, espesially Fe, and the good absorption of Fe in grape syrup, its consumption could offer potential benefits for improving human health. The results of our study support the hypothesis that grape syrup may have therapeutic effects on IDA.

### 4.1 Strengths and limitations

This study has several strengths. This study represents the first clinical trial to investigate the use of grape syrup, a readily available and a natural iron‐rich food product from Uremia and Iran, in treating IDA in women.

The study is limited by a relatively small sample size, a short intervention period, and a brief follow‐up duration.

## 5 CONCLUSION

Grape syrup significantly improved Hb, Hct, RBC, and MCV compared to placebo, suggesting its potential as a complementary therapy for IDA. Further research with larger sample sizes, longer follow‐up period was suggested for exploring long term efficacy and safety of grape syrup for IDA managment.

## AUTHOR CONTRIBUTIONS

**Rasul Pourhakim:** Data curation (equal); investigation (equal); methodology (equal); writing – original draft (equal). **Fatemeh Emadi:** Conceptualization (lead); data curation (lead); investigation (lead); methodology (equal); project administration (supporting); supervision (equal); visualization (lead); writing – review and editing (lead). **Saeed Razavi Dizaji:** Investigation (equal); methodology (equal); visualization (equal); writing – review and editing (equal). **Daryush Talei:** Formal analysis (equal); methodology (equal); validation (equal); writing – review and editing (equal). **Sayed Saeed Esmaeili Saber:** Conceptualization (equal); data curation (equal); investigation (equal). **Maryam Iranzadasl:** Investigation (equal); methodology (equal); writing – review and editing (equal).

## FUNDING INFORMATION

This study was supported by Shahed University (grant no. 22780).

## CONFLICT OF INTEREST STATEMENT

The authors declare that they have no known competing financial interests or personal relationships that could have appeared to influence the work reported in this paper.

## Data Availability

The data that supports the findings of this study are available on request from the corresponding author. The data is not publicly available due to privacy or ethical restrictions.
